# Impact of diet-derived signaling molecules on human cognition: exploring the food–brain axis

**DOI:** 10.1038/s41538-017-0002-4

**Published:** 2017-10-30

**Authors:** Raymond L. Rodriguez, John G. Albeck, Ameer Y. Taha, Kassandra M. Ori-McKenney, Gregg H. Recanzone, Tyler W. Stradleigh, Bronte C. Hernandez, Feng-Yao Vincent Tang, En-Pei Isabel Chiang, Lillian Cruz-Orengo

**Affiliations:** 1 Department of Molecular and Cellular Biology, College of Biological Sciences, One Shields Avenue, University of California, Davis, Davis, CA 95616 USA; 2 Department of Food Science and Technology, College of Agriculture and Environmental Sciences, One Shields Avenue, University of California, Davis, Davis, CA 95616 USA; 3 Department of Neurobiology, Physiology and Behavior, College of Biological Sciences, One Shields Avenue, University of California, Davis, Davis, CA 95616 USA; 4 Center for Neuroscience, College of Biological Sciences, University of California, Davis, Davis, CA 95616 USA; 5 Department of Psychiatry and Behavioral Sciences, School of Medicine, University of California, Davis, Davis, CA 95616 USA; 6 Department of Nutrition, China Medical University, Taichung, Taiwan; 7 Department of Food Science and Biotechnology, National Chung Hsing University, Taichung, Taiwan; 8 Agricultural Biotechnology Center, National Chung Hsing University, Taichung, Taiwan; 9 Department of Anatomy, Physiology & Cell Biology, School of Veterinary Medicine, University of California, Davis, Davis, CA 95616 USA

**Keywords:** Neuroscience, Systems biology, Dendritic excitability

## Abstract

The processes that define mammalian physiology evolved millions of years ago in response to ancient signaling molecules, most of which were acquired by ingestion and digestion. In this way, evolution inextricably linked diet to all major physiological systems including the nervous system. The importance of diet in neurological development is well documented, although the mechanisms by which diet-derived signaling molecules (DSMs) affect cognition are poorly understood. Studies on the positive impact of nutritive and non-nutritive bioactive molecules on brain function are encouraging but lack the statistical power needed to demonstrate strong positive associations. Establishing associations between DSMs and cognitive functions like mood, memory and learning are made even more difficult by the lack of robust phenotypic markers that can be used to accurately and reproducibly measure the effects of DSMs. Lastly, it is now apparent that processes like neurogenesis and neuroplasticity are embedded within layers of interlocked signaling pathways and gene regulatory networks. Within these interdependent pathways and networks, the various transducers of DSMs are used combinatorially to produce those emergent adaptive gene expression responses needed for stimulus-induced neurogenesis and neuroplasticity. Taken together, it appears that cognition is encoded genomically and modified by epigenetics and epitranscriptomics to produce complex transcriptional programs that are exquisitely sensitive to signaling molecules from the environment. Models for how DSMs mediate the interplay between the environment and various neuronal processes are discussed in the context of the food–brain axis.

## Introduction

Recent reports of adult hippocampal neurogenesis (AHN) in small animal models has stimulated interest in understanding the molecular mechanisms underlying this process and in particular, those genomic and environmental factors that influence neurogenesis in humans.^1–4^ The prospect that adult humans can improve neurological processes such as mood, emotion and cognition with food and lifestyle modification is intriguing. Research results on the role of physical activity,^5–7^ mastication,^8–10^ and cognitive training^11^ in promoting neurogenesis and cognitive performance are encouraging in that they may provide simple, noninvasive interventions for improving neurological health. The results of studies on the role of caloric restriction^12^ and food with all its diet-derived signaling molecules (DSMs) (e.g., macronutrients, micronutrients, biotransformed nutrients, phytochemicals, anti-nutrients and xenobiotics) are less clear but still promising.^12–14^ The problem of weak significance levels in studies on the impact of food and lifestyle on cognition was recently highlighted in a systematic evidence review entitled “Interventions to Prevent Age-Related Cognitive Decline, Mild Cognitive Impairment and Clinical Alzheimer’s-Type Dementia commissioned by the NASEM/NIA.”^15^ For this comprehensive meta-analysis, 263 eligible publications were chosen from a total 9448 references involving 13 different interventions. Of these 13 interventions, only four (physical activity, raloxifene—a selective estrogen receptor modulator, B vitamins and cognitive training) were associated with delaying or preventing age-related cognitive decline, albeit at low to moderate levels of statistical significance. The review mentioned several factors explaining the low statistical correlations between these interventions and cognitive health. These factors included, but were not limited to, large variations in study design, subject populations, protocols, as well as low adherence and high attrition rates. The lack of clear clinical endpoints, including different cognitive assessment tools, also confounded the interpretation of results and contributed to inconsistent study results. The NASEM/NIA review was less a critique of the role of food and DSMs on neurogenesis and cognition than it was a call for more, larger and better-designed studies including those involving multimodalities or “best packages” of interventions. The review also suggested that research on dietary interventions, including supplementation with B vitamins, should be a high priority.^15^

While the challenges of interpreting large numbers of dietary interventions in the aggregate are many, there remains a large body of literature describing the important and positive relationship between food and neurological development and function in young children^16–20^ and the elderly.^21–25^ In terms of the impact of diet on early brain development in children, Prado and Dewey^18^ concluded that undernourished children are at risk of not reaching their full developmental potential in cognitive, motor, and socio-emotional abilities. In many instances, however, these abilities can be restored if nutritional rehabilitation occurs early in brain development, including intrauterine brain growth. In the case of the aging brain, many molecular and cellular processes, including neural stem cell development, are altered in response to aging. Poor diet is known to cause age-related changes in the systemic environment that increases the risk of both chronic disease and cognitive decline among the elderly.^23^ The purpose of this review is to focus attention on the molecular mechanisms that transduce DSMs into the nonrandom, DNA-encoded, emergent adaptive transcription programs needed for neurogenesis and stimulus-induced neuronal, and synaptic, plasticity. Because of space limitations, we are unable to discuss in any detail the importance of epigenetics and the human microbiome on neurogenesis and cognitive function. Instead, we refer the reader to the following reviews on neuroepigenetics^1,4,26,27^ and the gut–brain axis.^28,29^

## The evolution of food–brain interactions

The wonders of the modern human brain can be traced to its humble beginnings. Starting with a brain of approximately 470 ml in the Hominini,^30^ the human brain has grown to about 1350 ml over the past 2 million years.^31^ The near tripling in the size of the human brain is the result of many factors not the least of which are the external inputs of energy and the molecular building blocks provided by macronutrients (e.g., proteins, carbohydrates and lipids). Although macronutrients are essential for energy and the assembly of neural and non-neural tissues, it is also likely that the micronutrients, biotransformed nutrients, phytochemicals and even anti-nutrients and xenobiotics (i.e., DSMs), triggered the plethora of molecular processes required for the growth, development and differentiation of the modern brain and all its parts. When stimuli from DSMs are integrated neuronally and subjected to the pressures of natural selection, cellular responses can emerge that produce higher-order cognition that is adaptive, sustainable and knowledge generating. This is almost assured considering the extensive feedforward/feedback regulatory controls at play in the brain as discussed later.

Since AHN was first discovered in the mammalian brain by Altman and Das,^32^ it was often considered a phylogenetic reversion, away from lifelong neurogenesis, in favor of neurological stability within the complexity of the brain.^33^ We now know that AHN occurs throughout the animal kingdom and while humans have fewer neurogenic zones than fish, within these neurogenic zones, substantial and highly functional neurons can be produced. This suggests that at least for the mammalian dentate gyrus, evolution has moved toward neurogenic plasticity rather than away from it.^33^ This idea is supported by recent studies using imaging connectomics^34^ and graph theory showing that normal brain maturation, from infancy to adulthood, involves significant co-evolution and integration of structural (neurons and glial cells) and functional (cognitive processes) networks.^35^ We propose that this co-evolution of neuronal and synaptic plasticity is supported, if not driven, by the constant interplay between the brain and external stimuli from food. This concept is consistent with the ecological intelligence hypothesis for primate brain evolution.^36^ According to this hypothesis, “foraging cognition” involving spatial memory, value-based decision making and inhibitory control creates those dynamic feedforward and feedback interactions that are adaptive and lead to higher-quality foods, more productive food sources and larger brains.^36,37^ We believe the principles of the food–brain axis described below, complement and extend the ecological intelligence hypothesis by connecting the nutritional environment with neurological structures and processes through well-studied signaling pathways and gene regulatory networks.

### The food–brain axis

In recent years, it has become apparent that all physiological, metabolic and genetic processes and systems are interconnected and interdependent to some degree. Examples include the inflammation–immunity axis,^38^ the hypothalamus–pituitary–gonadal axis^39^ and the gut–brain axis.^28,29^ In each case, an axis suggests diverse regulatory lineages, orchestrating control and outcomes over other critical processes. This makes all the systems involved highly sensitive to extracellular signals including those from the environment. The food–brain axis represents more than just connectivity and relatedness between what we eat and how our brain grows and functions; it illustrates dynamic interdependencies between food and neurological processes. This semi-quantitative interpretation of “axis” should enable researchers to categorize, quantify and predict neurological changes as a function of food quality and/or quantity.

**Fig. 1:**
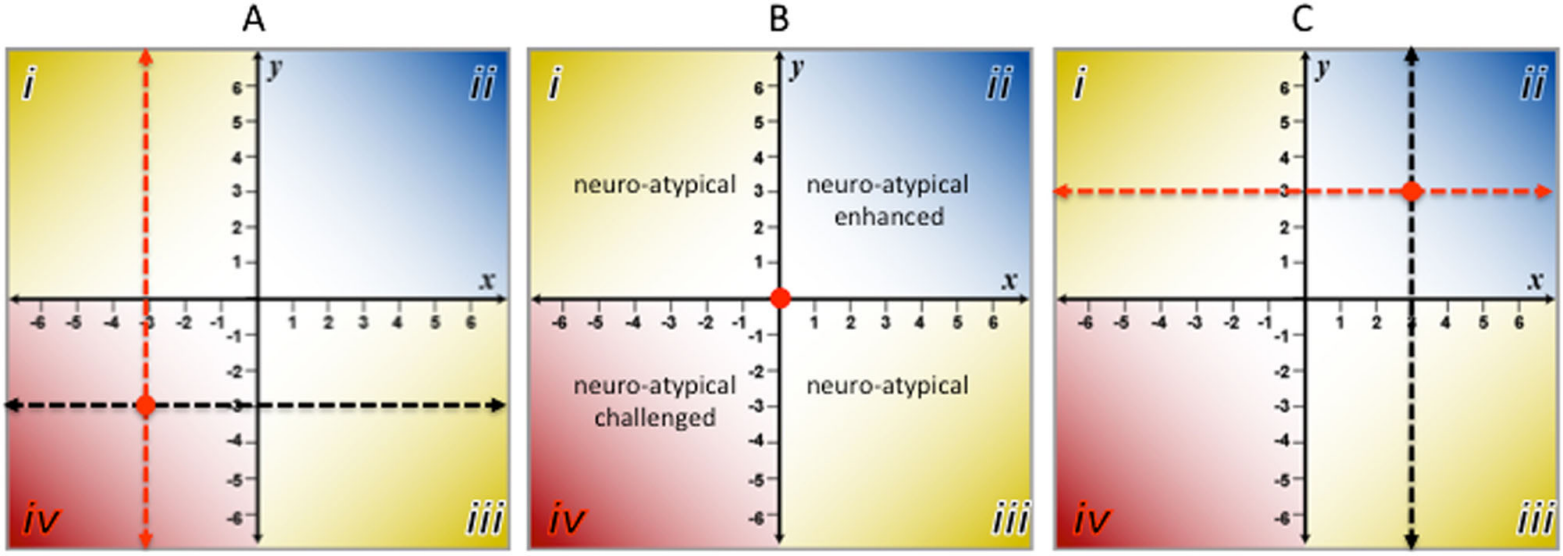
Four quadrants of the Food-Brain Axis. Three examples of the Food-Brain Axis where the X-axis represents increasing quantity and/or quality of dietary inputs (independent variables) and the Y-axis represent the changes in neuronal growth, development and cognitive function (dependent variables) from poor (-6) to optimal (+6) states. The transect points for the XY axes are represented by the red dots

Here, the food–brain axis is defined as a horizontal line of independent variables thought to be causative (e.g., food) that transects a vertical line of dependent variable thought to be the effects (e.g., neurogenesis, neuroplasticity and neuropathologies). Figure 1 shows three hypothetical examples (a, b and c) depicting the consequences of changing the quantity and/or quality of the food from poor (e.g., -3) to good (e.g., +3) on the *X*-axis, and its effect on neural growth, differentiation and function on the *Y*-axis. In addition to being an informative method for displaying the dynamic relationship between diet and brain structure and function, the food–brain axis organizes these interactions into four quadrants (i.e., i–iv). Figure 1b shows the nominal or neuro-typical condition for food–brain interactions whereas Fig. 1a-iv shows a neuro-atypical/challenged condition in which neurological processes are dysfunctional, degenerative and pathological as a result of poor diet. Conversely, Fig. 1c-ii illustrates a neuro-atypical/enhanced condition in which neurological processes and structures are enhanced in response to dietary inputs that are higher in quality and/or quantity.

The neuro-atypical quadrants A-i and C-iii pose interesting questions about food–brain interactions. In the case of quadrant A-i (neuro-atypical/enhanced) one might surmise that brain development and function (as defined by measures of intelligence) may be more dependent on robust genetic factors and age than on the quantity and/or quality of food intake. For example, it is known that the genetic contribution to human intelligence is approximately 80% for adults with additive genetic variance contributed by selective mating based on similar phenotypes.^40^ Therefore, if prenatal and early postnatal nutrition are adequate (e.g., nursing), the impact of nutritional deficiency later in life may have little or no measurable effect on cognitive performance. For quadrant C-iii, strong genetic determinants like Fragile X syndrome, Huntington’s disease, PKU as well as traumatic brain injury (TBI; Box 1) come into play. These are conditions that make neurological structures and processes refractory to the benefits of abundant, higher-quality dietary inputs. PKU, an autosomal recessive metabolic disorder, causes a toxic accumulation of dietary phenylalanine in the brain. If undiagnosed and left untreated, PKU can cause serious cognitive impairment, behavioral and mental disorders as well as seizures, regardless of food quality and/or quality. However, nutritional intervention with a low phenylalanine diet supplemented with large neutral amino acids, vitamin D and B12 can prevent or mitigate the physical, neurological and developmental problems associated with PKU.^41^ Therefore, interpreting the impact of food quality and/or quantity (*x*-axis) in quadrant A-i, age must be taking into account while in quadrant C-iii, early diagnosis of deleterious genetic factors or structural damage to brain tissues are key factors for consideration.

Food and Traumatic Brain Injury (TBI) The Centers for Disease Control and Prevention defines TBI as an injury that disrupts normal brain function.^42^ Injury includes bumps, blows or jolts to the head, penetrating trauma and explosive blasts. Even mild TBI can trigger neurodegenerative disorders.^42–44^ Most clinical studies typically monitor nutritional deficits as a consequence of TBI and only a handful of studies focus on diet as a factor in recovery.^43,45^ It should be noted that, morbidity and long-term mortality from TBI are associated with a decline in digestive function as a result of either multiple organ dysfunction or systemic inflammatory response syndromes.^46^ Animal studies are consistent with these clinical findings motivating the search for a more mechanistic approach to this question.^46^ Primary TBI (pTBI) is characterized by insult to neuronal membrane and vasculature. Vascular damage triggers secondary TBI (sTBI) involving a cascade of events starting with ischemia, production of ROS, further damage to neuronal membranes, increased intracellular Ca^2+^, endoplasmic reticulum stress and ultimately apoptosis and autophagy.^47^ The events associated with pTBI are so sudden that the impact of nutritional supplementation is difficult to assess. However, those dietary supplements thought to reduce the effects of sTBI are vitamin D with progesterone, antioxidant vitamins C and E, zinc, magnesium and the omega-3 fatty acid, docosahexaenoic acid (DHA).^44,48^ Interest in DHA is increasing because of evidence from animal studies suggesting that DHA has both preventive and therapeutic benefits against trauma and stroke.^5^ Among the mechanisms associated with DHA consumption or administration are 1) a reduction of neuronal and white matter loss, 2) decrease of proinflammatory cytokines, BBB leakage, matrix metalloproteinase activity and 3) regulation of microglia activation.^5^ Other food-based supplements frequently considered beneficial are glutamine, choline, creatinine, curcumin, flavonoids, phenolic acids, quercetin and resveratrol.^43,45,49–51^ Ketones metabolically derived from medium chain triglycerides or the high-fat ketogenic diet are also being considered for treating cognitive impairment present in many brain and metabolic disorders such as Alzheimer’s disease, trauma and the metabolic syndrome. Ketone bodies such as β-hydroxybutyrate can serve as energy substrates that compensate for deficits in brain glucose utilization, as seen in people with mild cognitive impairment, Alzheimer’s disease and insulin resistance.^52,53^

### DSMs and the brain

The impact of food on brain development and function has been extensively reviewed, although these reviews are often more descriptive than mechanistic in nature.^15,54,55^ They emphasize the neuroprotective aspects of nutrition but seldom discuss the mechanistic role of DSMs in AHN and cognition. Some of the first to systematically review research on the mechanistic effects of DSMs on the brain were Gomez–Pinilla^55^ and Zheng and Berthoud.^56^ A list of some of the dietary factors discussed in these reviews is shown in Table 1. While not an exhaustive list, Table 1 underscores the multifunctional character of DSMs that range from sources of energy and building blocks for cellular structures to chemical signals that trigger gene regulatory cascades that control transcriptional programs in the brain. One of the best examples of a multifunctional DSM is the long-chain polyunsaturated fatty acid (PUFA), DHA, which can serve as a nutrient, transcription regulator, immuno-modulator and neurotransmitter.

**Table 1:** Diet-derived signaling molecules (DSM) that supports brain function

DSM	Function in the Brain	References
Choline	A macronutrient important for normal brain development, nerve function; a precursor of acetylcholine which promotes cognitive flexibility and adaptive behavior in response to new and unexpected environmental circumstances	55,156
D-Glucose	Biotransformed from more complex sugars and carbohydrates; D-glucose provides the energy needs of the brain in the form of ATP; enhances cognitive function and neuroprotective for AD	78,107
Folate	Required for metabolism of 5-MTHF and homocysteine; deficiency in 5-MTHF is associated treatment-refractory depression while overproduction homocysteine is associated with neuropsychiatric disorders; folate is also a precursor for the methyl-donor, SAM, which is required to epigenetic modification of DNA and chromatin	21,55,131,136–138,149
Omega-3 fatty acids (EPA, DHA, ALA)	Neuroprotective against AD; reduces the levels of AD biomarkers (β-amyloid plaque and neurofibrillary tangles) in cerebral spinal fluid; DHA has been implicated reducing severity of depression and bipolar disorder	56–59,73,147,148, 157,158
Plant polyphenols	Neuroprotective for AD and Parkinson’s disease; neurotrophic and associated with enhanced neuronal survival and promotes neuronal differentiation in vitro; helps maintain metabolic homeostasis which has a protective effect on membranes; involved in histone deacetylation	13,43,45,49,54,55
Vitamin A	Antioxidant; prevents cognitive decline; perinatal deficiency correlated with increased risk of schizophrenia; promotes neuronal differentiation of neuronal stem cells	21,55,159
Vitamin B3 (niacin)	Transactivation of a PI3K/Akt signaling cascade to prevent/reduce brain damage from stroke; neuroprotective for Parkinson’s disease	156,160
Vitamin B6 (pyridoxine)	Coenzyme for the biosynthesis of neurotransmitters; required for metabolism of homocysteine which is implicated in the development of psychiatric disorders including depression	21,161
Vitamin B12	Essential for brain development, neuronal myelination and cognitive function including mood; methyl-donor for methionine and SAM, the latter serving as the methyl-donor for epigenetic modification of DNA and chromatin	15,131,162
Vitamin C	Neuroprotective against oxidative damage in the brain; higher intake associated with lower AD	21,55,163
Vitamin D	Neuroprotective against oxidative damage; deficiency correlated with greater risk of schizophrenia and multiple sclerosis	55,164
Vitamin E	Antioxidant; prevents membrane oxidation DHA peroxidation; slows cognitive decline and the advancement of AD	55,165

Table 1 lists twelve well-characterized DSMs and their purported and demonstrated impact on neurological function. Not shown are various non-dietary plant compounds (e.g., forskolin, huprazine A, ginko) and minerals (i.e., Ca, Cu, Fe, Se, Zn) known, or thought to be involved in preserving or stimulating cognition in humans and/or laboratory animals. Table adapted from Gomez-Padilla^55^

Reduced brain or circulating DHA concentration has been implicated in depression, bipolar disorder and attention deficit (AD) disorder.^57–59^ However, intervention studies with long-chain omega-3 PUFAs have yielded mixed results.^5,57–60^ One recent meta-analysis, however, suggested an overall beneficial effect for EPA in major depressive disorder patients, especially at high doses.^61^ Interestingly, the beneficial effects of EPA were also observed in subjects taking antidepressants. Whether the beneficial effects of high EPA dosage together with antidepressants are additive or synergistic can have significant therapeutic implications and thus requires further study. Most studies designed to assess the benefits of omega-3 PUFAs on children with AD disorder are inconclusive. Another recent study, however, showed significant improvement in working memory for children with attention deficit hyperactive disorder supplemented with EPA^62^ (see Box 2).

For DSMs to impact various neurological structures and functions in ways that produce neurogenesis, synaptic plasticity and adaptive behaviors, there must be an efficient communication system allowing dietary stimuli to be delivered to the brain from the gut. These connections are provide by the 400–600 million neurons in the human enteric system^77^ that creates a virtual information highway through which DSMs can communicate critical chemical information from the environment to the brain.^78^ Alternatively, oxygen and nutrients in peripheral blood can be delivered to the brain via the middle cerebral arteries and their fenestrated capillaries to support hippocampal^6^ and hypothalamic functions.^79^ These communication channels permit dietary inputs to be more than just fuel and building blocks for the brain, but also a means for delivering important chemical signals from the extracellular environment to the neuron where they are continually integrated into those signaling pathways and neuronal activity needed for metabolic homeostasis, cognition and overall health.^55,56,79^ Box 3, Box 4.

### Nucleic acid-based regulation of the food–brain axis

Central to our understanding of how the food–brain Axis functions is the premise that homeostasis and the adaptive behaviors associated with cognition are the result of the complex interplay between signal transduction and transcriptional programming in the neuron. Fortunately, evidence is mounting to support the notion that the brain’s cognitive and non-cognitive functions are encoded genomically^80,81^ and subject to epigenetic^1,82–84^ and epitranscriptomic^4^ modification. In the case of epigenetics, a recent study demonstrated that transient activation of mature neuronal circuits can up-regulate and down-regulate transcription, particular for early genes like *Arc*, *c-Fos* and *Jun-b*, by dynamic modification of chromatin accessibility (i.e., chromatin condensation or de-condensation).^27^ The authors of this study concluded that activity-induced reshaping of the transcriptome plays an important role in regulating synaptic plasticity, cognitive function and neurological disorders. Other studies show that activity-induced epitranscriptomics (i.e., post-transcriptional RNA editing and RNA methylation of coding and non-coding RNAs) in the brain can produce experience-dependent plasticity leading to learning and memory.^4,26,85^

One of the challenges to understanding a nucleic acid-based model for cognition is the temporal scales that can span several orders of magnitude between stimulus sensing and experience-dependent neuronal plasticity. For example, the time to elicit an electrophysiological signal from a neuron is in the range of microseconds, while the initiation of transcription and translation of RNA can span minutes to hours. On the other hand, the time required for learning and durable memory might take days to years.^85^ This temporal discordance makes the trajectory between stimulus sensing and neuronal plasticity non-linear and difficult to understand.

**Fig. 2:**
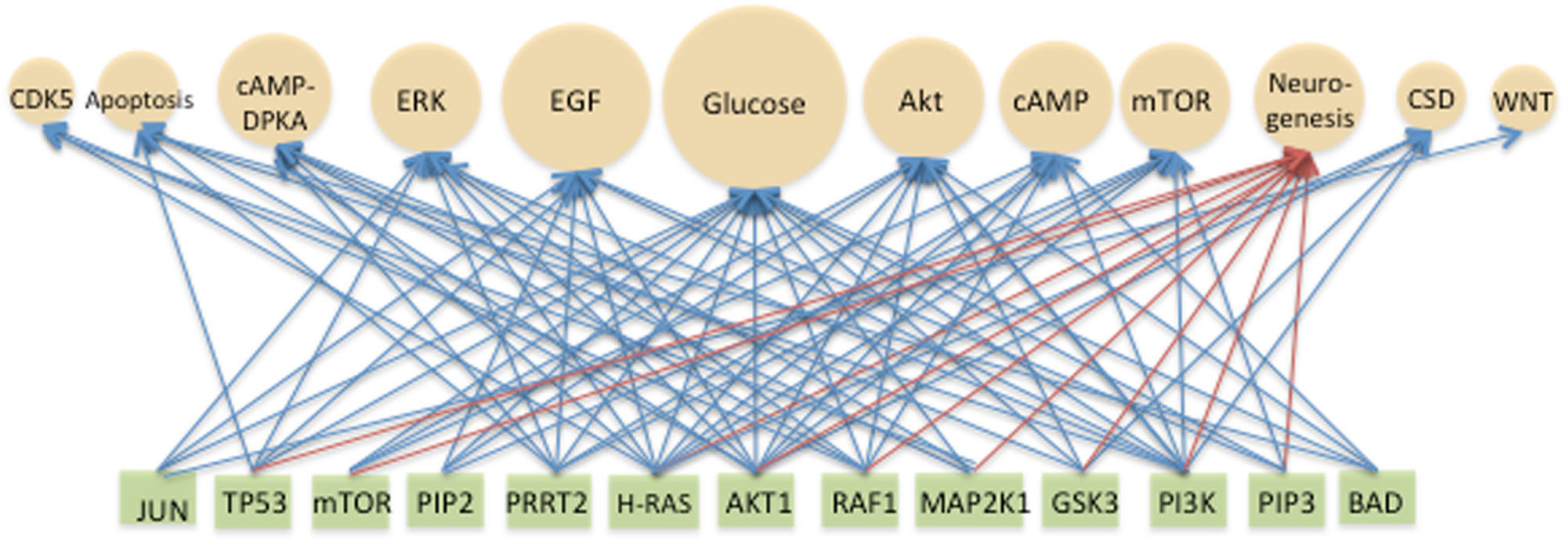
Transactivation of Signaling Pathways. A bipartite network illustrating the potential for transactivation (i.e., crosstalk) between 12 signaling pathways (beige spheres) and 13 signaling proteins (green rectangles). The glucose signaling pathway was used as a reference for weighting the other 11 signaling pathways in terms of their percent similarity with proteins involved in glucose signaling (sphere size approximates percent relatedness). The 13 signaling proteins were those common to glucose signaling and to at least three of the other signaling pathways (e.g., JUN is common to three pathways while AKT1 is common to ten pathways). Signaling proteins common to neurogenesis signaling are indicated by red arrows. The source of the signaling pathways and proteins was Pathways Online SABiosciences. For the neurogenesis signaling pathway, several sources were used.^56, 152–155^ A list of signaling pathways and proteins used to construct this network is provided in Supplemental Table 1. The selection of pathways and proteins was for illustrative purposes and not intended to be exhaustive. Abbreviations: Akt, serine/threonine kinase; cAMP, cyclic adenosine monophosphate; cAMP-DPKA, cyclic adenosine monophosphate-dependent protein kinase A; CSD, cytoskeletal dynamics; CDK5, cyclin-dependent kinase 5; EGF, epidermal growth factor; ERK, extracellular signal‐regulated kinase; mTOR, mammalian target of rapamycin; Wnt, wingless-integration site 1

One possible way to resolve these manifold differences in timing between sensing and response is to view them in the context of molecular processes that affect the rate and magnitude of each step along the trajectory. These molecular processes include epigenetic and epitranscriptomics modifications of chromatin, DNA and RNA (e.g., altered RNA structure, half-life, localization and ligand affinity and, methylation of histones, DNA and RNA^85–87^) and homeostatic scaling.^86,88^ All of these processes help create the diversity of transcription programs required for proper neuronal function. In the case of homeostatic scaling, both the proteome^88^ and transcriptome^86^ are altered to adjust synaptic strength up or down in response to changes in inputs. Deficiencies in homeostatic scaling are associated with neurological disorders such as autism spectrum disorder, epilepsy, Parkinson’s and schizophrenia and underscore the need for tight control over network activity for proper neuronal function.^88^ Interestingly, many of the transcription factors (e.g., CREB, Elk1, SRF), kinases (e.g., CaMK, CDK5, MAPK) and growth factors (e.g., BDNF) associated with homeostatic scaling are also components of pathways (e.g., ERK) that crosstalk with neurogenesis signaling (Fig. 2, Transactivation of Signaling Pathways, Supplemental Table 1 A comparison of signal transduction pathway relative of signaling for glucose signaling).^86^ In terms of the potential impact of inputs like DSMs on homeostatic scaling, expression of BDNF, a neurotrophic factor involved in neurogenesis, memory and learning, can be triggered by a variety of polyphenolics compounds found in plant-based foods (Table 2).

**Table 2:** Neurotrophic polyphenolic signaling molecules

Compound	Model	Pathway	Neurotrophic factors	Function	References
Astilbin	Mouse	Erk, Akt	BDNF	Antidepressant-like effects	166
Butein	Mouse	Erk, CREB	BDNF	Cognitive Enhancement	167
CAPE	Mouse	Nrf2/ARE	BDNF	Protective of dopaminergic neurons	168
Curcumin	Rat	Akt/GSk-3β	BDNF	Reduced β-amyloid-induced cognitive impairment	169
Fisetin	Mouse	Erk, CREB	BDNF	Cognitive enhancement	167
Resveratrol	Rat	Erk, CREB	BDNF	Antidepressant-like effects	170,171
Rosmarinic acid	Rat	Erk	BDNF	Antidepressant-like effects	172

Table 2 shows seven naturally occurring plant-based polyphenolic compounds and their impact on six different physiologically relevant signaling pathways and their neurotrophic effects on BDNF. Also, shown are the experimental animal models used to demonstrate these effects. (Adapted from Moosavi et al.^13^). Molecular weights (in kD) for these compounds are as follows: astilbin, 0.450; butein, 0.272; CAPE, 0.284; curcumin, 0.368; fisetin, 0.286; resveratrol, 0.228; rosmarinic acid, 0.360

*CAPE* caffeic acid phenethyl ester, *BDNF* brain-derived neurotrophic factor

Over the past decade, the toolbox for regulating gene expression in the mammalian brain has expanded to include long-noncoding RNAs,^89^ enhancer RNA^90^ and long-lived circular RNAs.^91^ These regulatory RNAs also have the potential to be modified by epigenetics and/or epitranscriptomics to regulate neuronal behaviors, synaptic scaling, plasticity and ultimately cognition.

The dynamics of the food–brain axis can also be bidirectional in that food intake can regulate the expression of genes involved in memory, learning and adaptive behaviors, while adaptive behaviors, learning and memory can regulate gene expression to change food intake. This neuronal bidirectionality is best illustrated by the peptide hormones leptin, ghrelin, insulin and nesfatin-1 that use nutrient sensing to regulate satiety, hunger and food reward signals in the brain.^92,93^ When expressed or injected into rat brains, for example, nesfatin-1 can create anorexia in rats by inhibiting food intake, modulating the excitability of glucose sensitive neurons, and acting on the melanocortin system to enhance UCP1 expression in brown adipose tissue.^93^ It is the bidirectional nature of activity-dependent changes in neuronal function that holds promise for dietary interventions designed to create those adaptive and positive self-managing behaviors that contribute to good mental health.

Lastly, in addition to epigenetics, epitranscriptomics and noncoding RNAs, many of the more traditional mechanisms come into play when discussing regulating gene expression in the brain. These mechanisms include negative and positive feedforward/feedback control^94,95^ of neuronal gene expression including the use of the same regulatory schemes that evolved over millions of years to support the normal growth and development of all physiological systems. These regulatory schemes include nutrient sensing, signal transduction and those *cis*-acting (e.g., promoters, terminators, and enhancers) and *trans*-acting (e.g., activators, repressors, coactivators and corepressors) elements required for transcriptional control in eukaryotes.^96^

### Nutrient sensing

The ability for cells to discern and respond to fluctuations in extracellular signals is required of all living cells and is the starting point for the homeostatic/allostatic control of all physiological systems. As previously discussed, nutrient availability is a strong selective pressure for shaping the evolution of most cellular processes.^36,97^ Pathways that detect extracellular and intracellular changes in levels of nutrients, biotransformed nutrients (e.g., glucose, vitamin D, vitamin A), energy and hormones, activate signaling pathways to compensate for these fluctuations and reestablish homeostasis. When food is abundant, nutrient-sensing pathways stimulate energy storage and anabolic processes while nutritional scarcity triggers homeostatic mechanisms that mobilize glucose and lipid stores^97,98^ by processes like autophagy.^97,99^ The glucose transport protein, GLUT2, is a good example of an evolutionarily conserved nutrient sensing mechanism. As a plasma membrane glycoprotein encoded by the *SLC2A2* gene in humans,^79^ GLUT2 can also be found in the brains of invertebrates like *Drosophila melanogaster.* ^100,101^ GLUT2 facilitates bidirectional glucose transport expressed in the pancreas and hypothalamus and because of its low affinity for glucose, it is considered an excellent glucosensor in astrocytes and neurons.^102^

### From glucose sensing to neurogenesis

Biological systems are parsimonious in terms of achieving adaptive responses to changes in the environment. As mentioned above, the brain draws upon efficient and well-developed molecular processes like nutrient sensing, transactivation of signaling pathways (i.e., signaling crosstalk) and stimulus-specific combinatorial gene regulation to support the neural growth, differentiation and experience-dependent neuronal plasticity.^56,103,104^ To see how transactivation of signaling is achieved one need only compare the components of various signaling pathways to a critically important pathway such as glucose signaling (Fig. 2).

As a nutrient, none is more important than glucose in terms of signaling changes in the extracellular environment and fueling cellular processes with ATP synthesis. In the brain, the energy generated from ATP hydrolysis is essential for sustaining ongoing neuronal activities as well as “housekeeping” functions (e.g., buffering ions, recycling neurotransmitters, and brain phospholipid remodeling that accounts for 20% of ATP consumption) in both the resting and awake brain.^105,106^ According to Zhu et al,^107^ the brain produces approximately 5.7 kg of ATP daily, which is equivalent to the complete oxidative combustion of 56 g of glucose in a single day. This provides 77% of the total energy needs of the cortical gray matter alone.^107^

As can be seen in Fig. 2, the potential for crosstalk between different signaling pathways is considerable. This suggests that any DSM, with even modest affinity for one of the signaling proteins in this limited list, has the potential to transactivate other signaling pathways to create interlocking pathways capable of linking neurogenesis to nutrient sensing. By using transactivation to interlock signaling pathways, even small fluctuations in the concentration of any component of a pathway can produce rapid, nonlinear and dynamical shifts in pathway outcomes.

### Polyphenolics: non-nutritive DSMs

While glucose may be the most essential of the DSMs, other macronutrients like amino acids^108^ and lipids^109^ can also activate signaling pathways. However, it is the non-nutritive bioactive compounds like plant-based phenolic acids that intrigue researchers as potential dietary interventions to support neurological health. The main reason for this interest is the excellent pharmacokinetic properties of many plant polyphenolics including their ability to cross the blood brain barrier (BBB).^54,110,111^ For example, a study using a noninvasive, localized BBB-opening technique in vivo, showed that molecules in the 3 to 70 kD could be easily delivered *trans*-BBB.^111^ Many polyphenolics fall in the range of 0.2 to 0.5 kD (Table 2). In another recent review of the consumption levels and neuroprotective properties of 17 phenolic acids, it was reported that the daily consumption of these compounds varied from country to country and by sex, with the highest consumption level among Europeans being 1786 mg/day for Danish men.^54^ Furthermore, ellagic acid, commonly found in many fruit and vegetable, was recently found to improve brain injury outcomes in rats and increase proliferation of neural stem cells through the Wnt/β-catenin signaling pathway.^112^ Other studies suggest that the antioxidant properties of polyphenolics can be neuroprotective for AD^113^ and modulate hippocampal neurogenesis.^114^ The mechanism for how this is accomplished is unclear, however, as shown in Table 2, expression of the neurotrophic factor BDNF can be induced by five different polyphenolics through one pathway (e.g., ERK) or by seven different compounds through six different pathways. That many of these pathways and their signaling proteins overlap with factors involved in neurogenesis signaling^114^ (Fig. 2), is good evidence in support of combinatorial crosstalk and its role in expressing a critical factor (i.e., brain-derived neurotrophic factor (BDNF) vital to learning, memory and higher order thinking.^114^

### Food–brain axis as a complex system

As discussed above, signaling molecules from the environment produce adaptive experience-dependent behaviors that make cognition a complex system. Complex systems in biology consist of many components with some degree of connectedness and interdependency. Guided by external input from the environment and a small set of rules (e.g., combinatorial and feedforward/feedback gene regulation and homeostasis) complex systems are capable of producing emergent adaptive behaviors^125,126^ that are not observed in random networks. Furthermore, these adaptive behaviors emerge dynamically and do not adhere to linear, dose-dependent or deterministic kinetics. Fortunately, these behaviors are stochastic and therefore, they can be described probabilistically.^125^ One of the best descriptions of a complex biological system is that of Gregor et al,^127^ who, in discussing the “Onset of Collective Behavior in Social Amoebae,” described signaling as a dynamical process capable of self-organizing the behavior of individual cells into synchronous pules of cAMP synthesis in a population of cells. This has led some to posit that complexity is a fundamental property of nature that guides growth and behavior of all living cells.^128^

Essential to producing emergent adaptive behaviors by neurons is the requirement for large amounts of informational input (i.e., DSMs) from the environment^126,129^ and a “decision space” where inputs are turned into outputs (i.e., solutions). Because of the large number of decisions that must take place in a complex system like the brain, input must be abundant and diverse. In the context of the food–brain axis, this means that dietary input should be rich in non-nutritive bioactive molecules (e.g., polyphenolics) as well as nutritionally dense macronutrients (e.g., carbohydrates, amino acids, omega-3 PUFAs). As shown in Fig. 1, changing the quality and/or quantity of dietary input can change the set point in the food–brain Axis to produce experience-dependent changes in the brain that are either advantageous or deleterious to cognitive health.

PUFAs and the Brain Phospholipids make up 99% of the brain’s esterified fatty acids. Approximately 20% of fatty acids bound to phospholipids consist of the long-chain polyunsaturated fatty acids (PUFAs) arachidonic acid (AA, 20:4n-6) and DHA (22:6n-3), which preferentially incorporate to the stereospecifically-numbered (sn-2) position of the phospholipid molecule.^63,64^ AA and DHA account for approximately 87 and 68% of total brain PUFAs in rats and humans, respectively, reflecting selective mechanisms for incorporating and retaining them within brain membrane phospholipids.^63,64^ Synthesis of AA and DHA in the brain is very low (<1%),^65,66^ so they must be obtained from circulating AA and DHA derived directly from the diet or synthesized in the liver from dietary linoleic (LA, 18:2n-6) and alpha-linolenic acid (ALA, 18:3n-6), respectively.^67,68^ AA and DHA passively cross the BBB in their unesterified form.^69^ A recent study showed that the incorporation rate of unesterified DHA by the brain exceeds that of esterified DHA by 10-fold, confirming that the unesterified plasma pool is the main source of DHA (and other fatty acids) to the brain.^70^ In the brain, AA and DHA regulate transcription and neuroreceptor-coupled signaling^71,72^ and serve as precursors to bioactive lipid mediators that modulate immunity.^73,74^ The brain may be more vulnerable to low DHA than AA, because LA, the dietary precursor to AA, exceeds ALA in food by approximately 10-fold.^75,76^

Transactivation, feedback loops and complexity While reports on the ability of DSMs to impact neural development and function are many, it is more challenging to identify the signaling proteins through which their influence is exerted. This is due in part to the complexity of signal transduction networks regulating neuronal identity and activity. Two of the best examples of this complexity are the transcription factors CREB and c-Fos, which play essential roles in long-term modulation of neuronal activity.^115^ A series of signaling pathways including cAMP/PKA, Ras/ERK, Ca^++^/CaMK, and PI3K/Akt converge to regulate *CREB* and *c-Fos* gene expression.^115,116^ This combinatorial control allows neurons to make long-term changes in their activity profile in response to both membrane depolarization and hormonal inputs. However, recent work in other cell types has revealed that this core set of pathways is also integrated with metabolic signaling. The Ras/ERK cascade is mutually antagonistic with AMPK, a kinase that becomes active when cellular ATP levels fall, through both regulatory phosphorylation^117,118^ and through mutual interactions with the scaffold protein KSR2.^119^ Similarly, PI3K/Akt signaling is tightly intertwined through both positive and negative feedbacks with the mTOR kinase complexes,^120^ which are highly sensitive to amino acid availability.^121^ Beyond these interconnections, this network can be expanded to include many more components and feedback loops.^122^ Thus, macronutrient availability presumably influences the decision of neurons to activate *CREB* and *c-Fos*, and thereby alter their activity profile. This implication has yet to be explored in detail but an emerging body of research suggests that DSMs do indeed impact the CREB and c-Fos signaling networks in the neuron.^123,124^ The elaborate feedback structures of these networks create a formidable barrier to understanding signaling in the neuron and necessitate a systems-level approach to unambiguously identify the molecular mode of action for DSMs.

1-Carbon metabolism and neurological function Perturbations in 1-carbon (1C) metabolic pathways significantly affect brain health and neurological functions. In a rat hippocampal cell line, folate deficiency was shown to cause differentiation-associated apoptosis, homocysteinylation and subsequent aggregation of neuronal proteins that contributes to alterations of differentiation and plasticity.^130^ Folate/vitamin B12 deficiency during gestation and lactation impairs cerebellar synapsin expression through a deregulation of ER-α//Src tyrosine kinase pathway in rats.^131^ In humans, the accelerated rate of brain atrophy in the elderly with mild cognitive impairment can be alleviated with vitamin B-complex supplementation.^132^ Interventions with folinate, a folate coenzyme, helps stabilize treatment for schizophrenia associated with folate receptor autoantibodies.^133^ Maternal dietary choline regulates development of the cerebral cortex in the offspring mice.^134^ while excess methionine inhibits neural tube closure in mouse embryos.^135^ Key enzymes in 1C metabolism are also closely related to brain health and neuro-functions. One example is the enzyme glycine N-methyltransferase, (GNMT) which plays critical roles in folate dependent reactions,^136^ methyl group homeostasis,^137^ and cellular defense against DNA damage.^138^ It is plausible that this enzyme is also important for proper neuronal function. In fact, enhanced expression of GNMT in cortical mixed neuron-glial cultures culture has been shown to be neuroprotective.^139^ In GNMT knockout mice, alterations in the adenosylmethionine pathway impair neurogenesis and contribute to cognitive decline.^140^ Mutations in genes encoding the glycine cleavage system also predispose mice and humans to neural tube defects.^141^

## Conclusion

Studies on the impact of food on neurological health are advancing our understanding of the dynamic interactions of the food–brain axis. This is important as the proportion of elderly (65 and older) in the global population with AD is expected to reach 70 million by the year 2030.^142,143^ It is not surprising that many individuals are anticipating the development of preventions, treatments and cures for neurodegenerative and neuropsychiatric diseases based on novel therapeutics and dietary interventions involving the foods we eat daily. This heightened expectation is fostered by research (predominantly involving small laboratory animals) demonstrating that many foods contain compounds that are neuroprotective, antipsychotic and anti-depressive (Tables 1, 2). It should be stressed, however, that mental and physical health are tightly linked and both can be strongly influenced by genetics and environmental factors such as physical activity and nutrition. In the case of AD, several genetic factors have been identified including the ε4 allele of the *APOE* gene, which is a strong risk indicator for both AD and coronary artery disease.^144,145^ Both diseases are also associated with sedentary lifestyle and obesity.^143,146^ Therefore, the expectation that there will be the nutritional equivalent of a “magic bullet” to ensure normal cognitive function into old age may be premature. Currently, PUFAs like DHA and EPA are being used in psychotherapy for AD,^73^ depression^147^ and personality disorders.^148^ Although PUFA responsiveness in all of these studies was slight to modest, the results are encouraging. Another recent study showed that a cerebral folate deficiency involving 5-MTHF is likely responsible for treatment-refractory depression and that subjects receiving sapropterin, a tetrahydrobiopterin analog, showed marked improvement.^149^

Finally, strategies for enhancing cognition and therapies for treating neuropathologies that include evidence-based nutrition should become more common as we learn more about the food–brain axis. This will require a multiscale, top-down approach that includes diverse data sets that span different locations, scales (e.g., tissues, cells and molecules) and time points to reveal the underlying connectivity, interdependencies and adaptiveness of biological systems like the brain.^150,151^ From nutrient to nucleotide to neuron, all scales must be analyzed vertically and orthogonally to fully understand the molecular basis of cognition in all its forms and dysfunctions. Accomplishing this daunting task will require collaboration and communication across multiple disciplines like food science, nutrition, genomics, molecular biology, neuroscience and informatics. Such transdisciplinary approaches are not only vital to understanding food–brain axis but other complex biological systems as well. Recognizing the importance of DSMs in signaling those transcription programs needed for neurogenesis and activity-dependent changes in neuronal functions is an important first step.

## Electronic supplementary material

Supplemental Table 1

## Electronic supplementary material

**Supplementary information** accompanies the paper on the *npj Science of Food* website (10.1038/s41538-017-0002-4).
